# HGF alleviates septic endothelial injury by inhibiting pyroptosis via the mTOR signalling pathway

**DOI:** 10.1186/s12931-020-01480-3

**Published:** 2020-08-14

**Authors:** Fei Peng, Wei Chang, Qin Sun, Xinyi Xu, Jianfeng Xie, Haibo Qiu, Yi Yang

**Affiliations:** Jiangsu Provincial Key Laboratory of Critical Care Medicine, Department of Critical Care Medicine, Zhongda Hospital, School of Medicine, Southeast University, 87 Dingjiaqiao Rd, Nanjing, 210009 People’s Republic of China

**Keywords:** Sepsis, Endothelial injury, Pyroptosis, HGF, mTOR, Mitochondria physiology

## Abstract

**Background:**

Endothelial injury is one of the predominant pathophysiological characteristics of sepsis and is the major cause of sepsis-induced multiple organ failure. Endothelial pyroptosis is a fatal mechanism of endothelial injury in sepsis, and specific, effective therapies are lacking. Although hepatocyte growth factor (HGF) has been shown to have anti-apoptotic and anti-necrotic effects, whether it prevents pyroptosis to improve endothelial injury in sepsis remains unclear.

**Methods:**

Recombinant HGF was intravenously injected into mice with sepsis caused by caecal ligation puncture (CLP). Histopathological examination and transmission electron microscopy (TEM) were used to measure lung vascular endothelial injury. Lipopolysaccharide (LPS) was transfected into EA.hy926 cells to induce endothelial pyroptosis, and the cells were treated with HGF in the presence of inhibitors of c-Met and mTOR, namely, PHA-665752 and rapamycin, respectively. The mTOR signalling pathway and mitochondrial physiology were assessed using Western blot and flow cytometry.

**Results:**

Intravenous HGF effectively alleviated pulmonary vascular endothelial injury and acute lung injury in the septic mice. The TEM results of lung tissue revealed that HGF attenuated pulmonary vascular endothelial pyroptosis, which was confirmed in vitro. Transfected LPS induced the pyroptosis of EA.hy926 cells and damaged their paracellular permeability, and these effects were ameliorated by treating the cells with recombinant HGF. The protective effect of HGF against pyroptosis was dependent on c-Met/mTOR signalling. mTOR activation effectively protected mitochondrial physiology and decreased reactive oxygen species (ROS) production in EA.hy926 cells in vitro.

**Conclusions:**

These results demonstrated that HGF protected mitochondrial physiology by activating mTOR signalling to partially ameliorate endothelial pyroptosis and attenuate vascular endothelial injury and acute lung injury in sepsis animal model.

## Introduction

Sepsis is a common clinical syndrome defined as life-threatening organ dysfunction caused by a dysregulated host response to infection [1]. Although numerous advances in management and treatment have been implemented to battle sepsis, the overall mortality rate remains as high as 35–45% [2, 3]. An in-depth exploration of novel therapeutic targets or strategies for sepsis is urgently needed.

One of the most important pathophysiological hallmarks of sepsis is vascular endothelial injury [4, 5], which rapidly results in tissue oedema and inflammation spread and is responsible for the progression of multiple organ failure during sepsis [5]. Although anticoagulant therapy and nitric oxide synthase inhibitors have shown protective effects in the endothelium, effective treatments for endothelial cell injury in sepsis that improve prognosis are lacking [6]. Therefore, it is of great significance and translational value to explore mechanisms and novel therapeutic options of endothelial cell injury.

Hepatocyte growth factor (HGF) is a pleiotropic cytokine involved in multiple cellular and biological processes, including improvement of cell injury and alleviation of inflammation. Previous studies demonstrated that lipopolysaccharide (LPS)-induced organ injury in rodents and that plasma HGF concentrations were increased in patients with systemic inflammatory response syndrome and early-phase sepsis [7, 8]; these increase of HGF are suggested to serve a compensatory mechanism to minimize LPS-induced cell and organ injury. Our previous studies found that mesenchymal stem cells (MSCs) with high HGF expression and secretion protect the pulmonary endothelial cell monolayer against LPS-induced hyperpermeability and monolayer integrity disruption and subsequently alleviate LPS intratracheal instillation-derived acute lung injury in rats [9, 10]. Although HGF was reported to have anti-inflammatory effect on endothelial and protect the endothelial barrier in vitro, the molecular mechanisms underlying the protection against septic endothelial injury by HGF remain unclear.

Endothelial pyroptosis is a vital characteristic of septic endothelial injury. Pyroptosis is a recently recognized form of inflammatory programmed cell death that is different from apoptosis and necrosis in terms of molecular mechanisms and cellular representation; pyroptosis plays a critical role in the progression of sepsis [11]. Intracellular LPS or damage signalling activate inflammatory caspases and initiate pyroptosis, which is executed by gasdermin pores and ends with cell dissolution, with a massive release of damage-associated molecular patterns (DAMPs) [12]. Large amounts of endothelial pyroptosis destroying the endothelium were observed in the lungs and kidneys of septic mice [13, 14]. Although a small amount of evidence has revealed that HGF exerts antiapoptotic and cytoprotective effects in various epithelial and endothelial cells, e.g., HGF attenuates LPS-derived endothelial apoptosis [15], modulates chemotherapeutic agents induced autophagy and necrosis [16], whether HGF protects the vascular endothelium against pyroptosis in sepsis remains unknown.

This study investigated the role of HGF in the improvement of septic endothelial injury and the underlying mechanism. With the use of animal models, imaging studies, biochemical assays, and molecular inhibition approaches, we show that HGF ameliorates septic endothelial pyroptosis in vivo and in vitro and that the mammalian target of rapamycin (mTOR) signalling pathway plays a central role in this process.

## Materials and methods

### Reagents

Dulbecco’s modified Eagle’s medium (DMEM), foetal bovine serum (FBS), bovine serum albumin (BSA), recombinant HGF, 6-diamidino-2-phenylindole dihydrochloride (DAPI), and propidium iodide (PI) were obtained from Gibco (Grand Island, NY, USA). Lipopolysaccharide from *Escherichia coli*, serotype O127:B8, and penicillin/streptomycin were obtained from Sigma (St. Louis, MO, USA). The Lipofectamine 2000 reagent was purchased from Invitrogen (Carlsbad, CA, USA). Rapamycin and PHA-665752 were obtained from MCE (NJ, USA). The LDH cytotoxicity assay kits were from Beyotime Biotechnology (Nanjing, China). Interleukin (IL)-1β, IL-18 and tumour necrosis factor (TNF)-α enzyme-linked immunosorbent assay (ELISA) kits were purchased from ExCellBio (Shanghai, China). The rabbit monoclonal phospho-Met antibody (Tyr1234/1235) (#3077), rabbit monoclonal Akt (pan) antibody (C67E7) (#4691), rabbit monoclonal phospho-Akt (Ser473) antibody (#4050), rabbit monoclonal mammalian target of rapamycin (mTOR) antibody (#2983S) were from Cell Signaling Technology (Beverly, MA, USA), and the rabbit monoclonal mTOR (phospho S2448) antibody (ab109268), rabbit monoclonal Met (c-Met) antibody (ab51067), and recombinant rabbit monoclonal pro Caspase-1 + p10 + p12 antibody (ab179515) were from Abcam (Cambridge, England, UK). The mouse monoclonal antibodies against gasdermin D (GSDMD) (sc-393,581) and β-actin (sc-517,582) were obtained from Santa Cruz Biotechnology (Santa Cruz, CA, USA). The HRP-conjugated IgG antibodies were from ZSGB-BIO (Beijing, China). All the other chemicals used in this study were of analytical grade and were obtained from Sigma (St. Louis, MO, USA) or Beyotime Biotechnology (Nanjing, China) unless otherwise stated.

### Cell culture and treatment

The human umbilical vein cell line (EA.hy926 cells) was cultured with DMEM containing 10% FBS at 37 °C for 24 h in 5% CO_2_ and 95% air. At the end of the incubation, the cells were incubated with serum-free medium for 1 h, and the culture grew to 80% confluence prior to the initiation of the experimental treatments. The cells were cultured in 6-well plates with 2 mL of DMEM containing 10% FBS and transfected with 2.5 μg/mL LPS using 2 μL/mL Lipofectamine 2000 (Lipo2000). Then, the stimulated cells were treated with 25 ng/mL HGF immediately and 6 h later, with or without 50 nM PHA-665752 or 20 nM rapamycin.

### Mice

C57BL/6 J (B6) mice were bred and maintained under specific pathogen-free conditions at Southeast University. Six- to 8-week-old mice were subjected to caecal ligation and puncture (CLP). Mortality was assessed every 3–4 h. In some sepsis treatment experiments, 1 μg/g HGF was subsequently intravenously administered to the mice via the tail vein immediately and at 12 h after the CLP operation [17]. As a control, equal amounts of normal saline were administered in the same manner. To ensure that all regions were analysed with equal probability, an unbiased sampling cascade, systematic uniform random sampling (SURS), was applied, as recommended by the European Respiratory Society and the American Thoracic Society [18]. This study followed the national guidelines and protocols of the National Institutes of Health and was approved by the Local Ethics Committee for the Care and Use of Laboratory Animals of Southeast University.

### Evans blue pulmonary transvascular flux measurements

We performed an Evans blue extravasation assay to measure vessel endothelial permeability. Briefly, Evans blue (20 mg/kg) in 1 mL saline was injected into the mice and allowed to circulate in the blood vessels for 1 h. Intravascular Evans blue was washed by heparinized normal saline perfusion from the right ventricle for 2 min. The mouse lungs were excised, weighed, homogenized in 1 mL PBS, and extracted overnight in 2 mL formamide at 60 °C. The Evans blue concentration in the lung homogenate supernatants was quantified by the spectrophotometric method at absorbances of 620 and 740 nm. The lung weight/body weight (LW/BW) ratio was calculated to measure the pulmonary oedema.

### Lung histopathology

For histopathological examination, the lung tissues were harvested, fixed in 4% paraformaldehyde for 24 h, embedded in paraffin and cut into 4-μm-thick sections, followed by H&E staining. The sections were scanned by microscopy, and the lung injury score was quantified based on the images of 6 randomly chosen fields, following five criteria: oedema, alveolar and interstitial inflammation, alveolar and interstitial haemorrhage, atelectasis, and hyaline membrane formation. Each criterion was graded according to a 5-point scale (0–4) [19], more details about the scales are in the Additional file 1. The total lung injury score was calculated as the sum of the five criteria.

### TEM examination

For transmission electron microscopy (TEM) examination, lung tissues and stimulated cells were harvested and cut into < 1-mm^3^ sections and fixed in ice-cold 2.5% glutaraldehyde for 2 h. Subsequently, the samples were postfixed in 1% osmium tetroxide for 1 h, dehydrated through an ethanol series (50, 70, 95 and 100%) and embedded in epoxy resin. Finally, the ultrathin sections (60–80 nm) were double-stained with uranyl acetate and lead citrate and examined via TEM (Hitachi HT7700, Tokyo, Japan). For the lung tissue sections, the vascular endothelial cells were scanned, and the ultrastructural details of the pyroptotic endothelial cells were analysed. For the cell sections, the cells were randomly selected to measure mitochondrial injury by a blind observer.

### Flow cytometry

For the mitochondrial physiology assay, ROS levels and mitochondrial activity were measured by flow cytometry. EA.hy926 cells were treated as described, stained with DCFH-DA and MitoTracker for 30 min, and then analysed with flow cytometry. Data were acquired from 50,000 events using a BD LSR Fortessa (BD Biosciences), and the data were analysed by BD FACSDiva (BD Biosciences). The percentage of ROS-FLICA- or MitoTracker-APC-stained cells was analysed.

### Immunofluorescence

EA.hy926 cells cultured on glass coverslips were treated as described above and washed with PBS. After staining with MitoTracker for 30 min and washing with PBS, the cells were stained with DAPI for nuclear counterstaining. The stained slides were photographed using a fluorescence inversion microscope system (Olympus, Tokyo, Japan), the cells were randomly selected to measure mitochondrial injury.

### Western blot

EA.hy926 cells were cultured in 6-well plates and treated according to the experimental design described above. Then, whole cell lysates were harvested from the EA.hy926 cell monolayers. The total protein was quantified using the BCA method and adjusted to equal amounts. The protein mixtures were separated via 10% SDS-PAGE and transferred to active polyvinylidene difluoride membranes. After the transfer, the membranes were blocked with 5% BSA-TBST for 1 h at room temperature and then probed with primary antibodies against caspase-1 (1:200), GSDMD (1:1000), Met (1:1000), p-Met (1:1000), AKT (1:1000), p-AKT (1:1000), mTOR (1:1000) and p-mTOR (1:1000) at 4 °C overnight. The membranes were probed with an anti-β-actinantibody (1:1000) to control for protein loading and then incubated for 2 h at room temperature with HRP-conjugated secondary antibodies (1:1000). The results were scanned using a gel imaging system (UVP Company, Upland, CA, USA). Densitometry measurements were performed with Image Lab software (Bio-Rad Laboratories, Hercules, CA, USA).

### LDH release

EA.hy926 cells were cultured overnight in 96-well plates and transfected with 2.5 μg/mL LPS using Lipofectamine 2000 reagent for 12 h. The culture medium was collected and analysed using an LDH cytotoxicity assay kit according to the manufacturer’s instructions. The absorbance was measured at a wavelength of 490 nm. The LDH release of each sample well was calculated by dividing the positive well after subtracting the negative well.

### Elisa

EA.hy926 cells were cultured overnight in 24-well plates and transfected with 2.5 μg/mL LPS using Lipofectamine 2000 reagent for 12 h. The culture medium was collected and analysed by IL-1β and IL-18 ELISA kits according to the manufacturer’s instructions. The absorbance was measured at a wavelength of 450 nm. The concentrations of the cytokines in each sample well were calculated based on a concurrent standard curve.

### Statistical analysis

The data are expressed as the mean ± standard deviation on the basis of at least three separate experiments. The data were analysed using SPSS version 23.0 (SPSS Inc., Chicago, IL, USA). Significant differences amongst the mean values of multiple groups were evaluated with one-way ANOVA followed by Student-Newman-Keuls’ method. Survival was analysed using the log-rank test. A two-sided*P* value < 0.05 was considered statistically significant.

## Results

### HGF effectively alleviated acute lung injury in sepsis

**Fig. 1:**
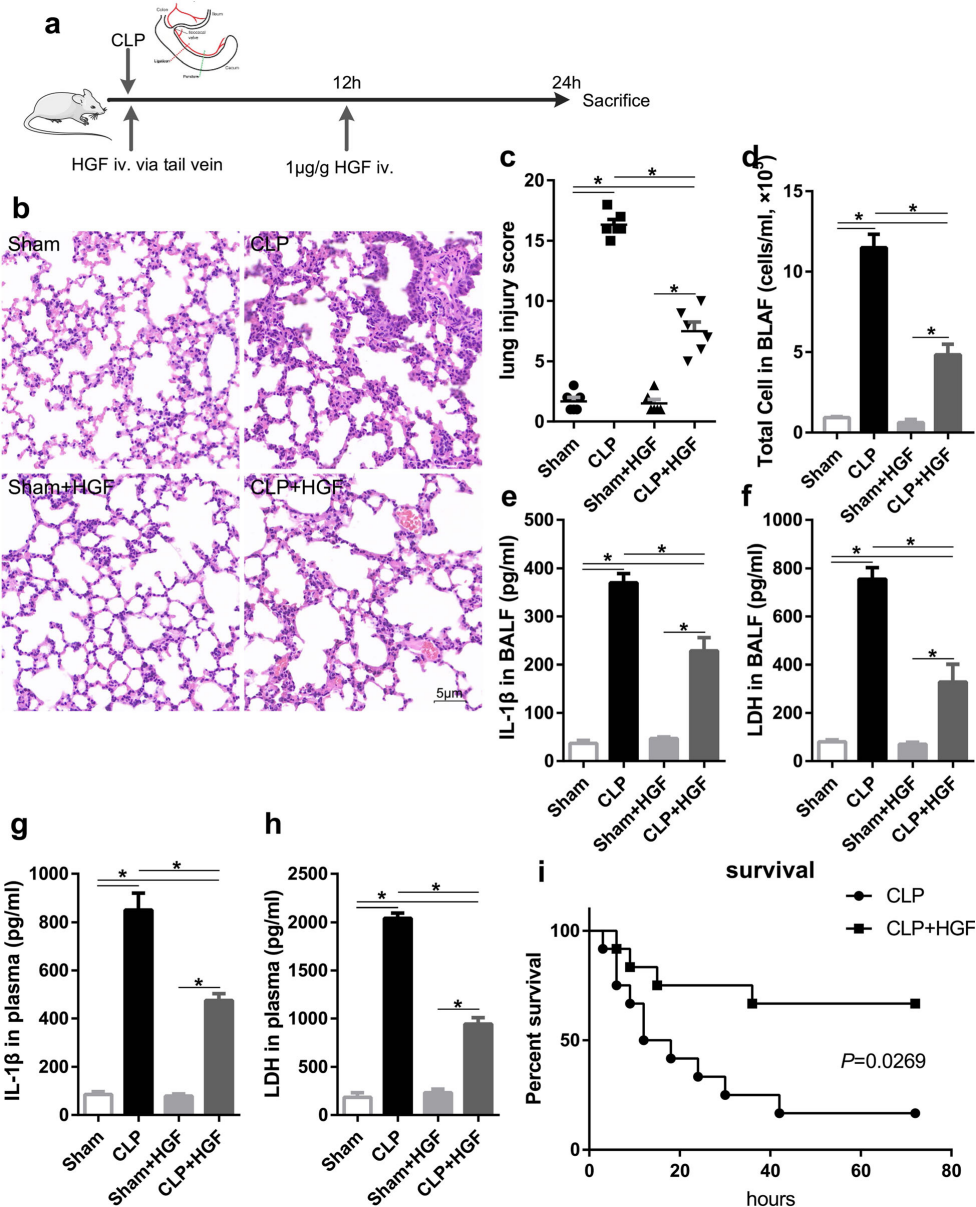
HGF alleviated acute lung injury in sepsis. C57BL/6 J mice were randomly assigned to 4 groups. **a** The HGF treatment schedule, recombinant HGF (1 μg/g) was intravenously administered to the mice via the tail vein immediately and at 12 h after the operation. The mice were sacrificed 24 h after the operation. **b** Lung histopathological features; **c** Lung injury score, six random fields in a section from each mouse were photographed and assessed; **d** Total cell number in the BALF (cells/ml, × 10^5^) was counted by Cell Counter; **e, f** IL-1β and LDH in the BALF (pg/ml) were measured by ELISA; **g, h** IL-1β and LDH in the plasma (pg/ml) were measured by ELISA; *n* = 3, *P < 0.05; (I) Mortality was assessed every 3–4 h, the survival was monitored until 72 h, and the survival curve was analysed, *n* = 12

To explore the effect of HGF on acute lung injury (ALI) in sepsis, we intravenously injected recombinant HGF into mice with sepsis caused by caecal ligation puncture (CLP) (Fig. 1a). Histopathological examination showed severe inflammatory cell infiltration, alveolar injury, interstitial oedema, and alveolar collapse in the lung tissues from the septic mice compared with those from the sham mice 12 h after the procedure (Fig. 1b). Compared to CLP alone, HGF treatment dramatically abated the inflammation and alleviated the lung injury induced by polymicrobial sepsis (Fig. 1c-d). With the increased invasion of a plethora of immunocytes, a surge in cytokines, such as IL-1β, IL-18, and lactate dehydrogenase (LDH), was observed in the plasma and bronchoalveolar lavage fluid (BALF) from the septic mice in the CLP group; however, this effect was abrogated by intravenous administration of HGF in the CLP + HGF group (Fig. 1e-h). In addition, the administration of recombinant HGF significantly reduced the mortality of the septic mice (Fig. 1i). There was no significant difference between the Sham and Sham+HGF groups.

### HGF attenuated pulmonary vascular endothelial injury in septic mice

**Fig. 2:**
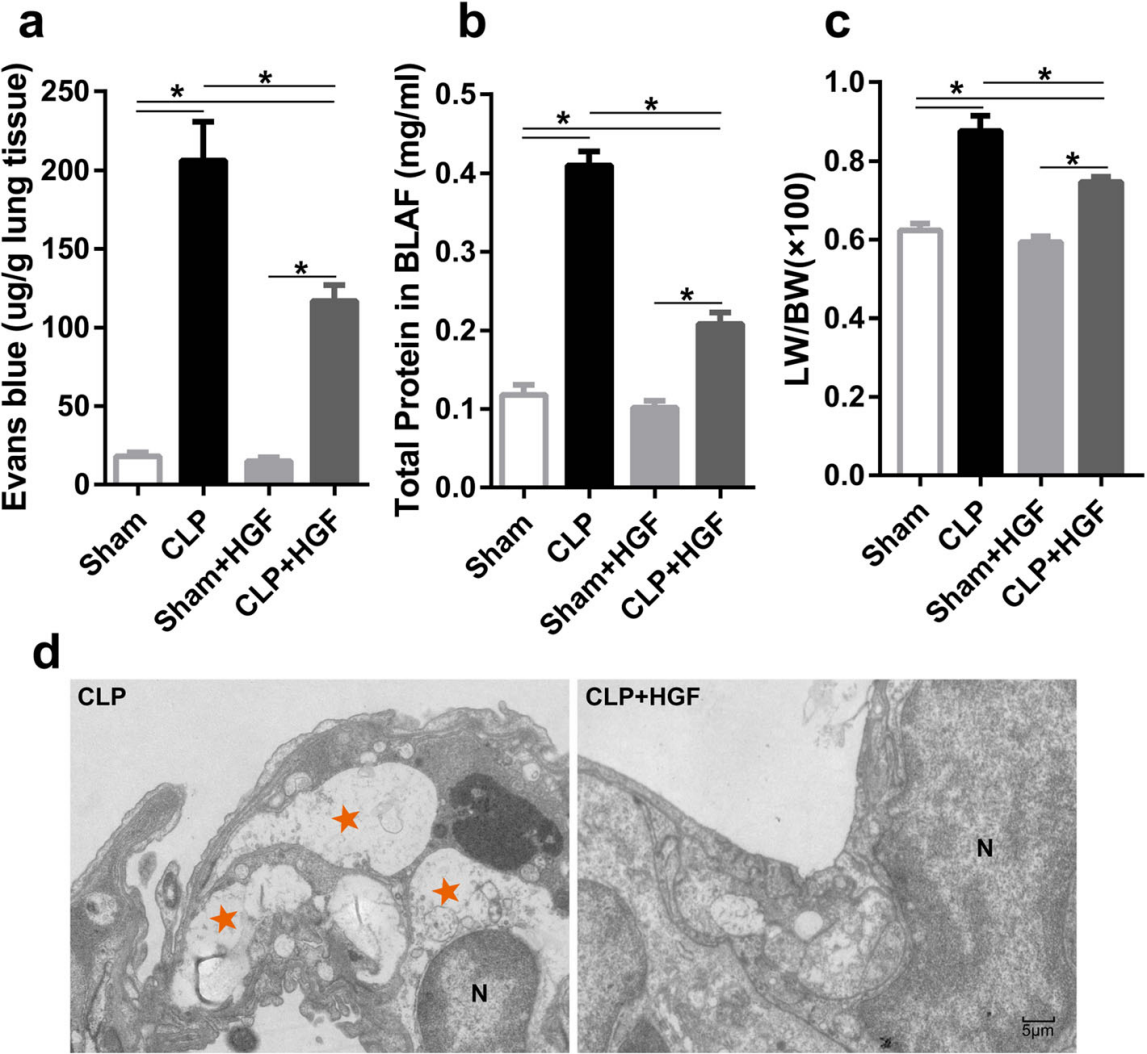
HGF attenuated pulmonary vascular endothelial injury in septic mice. **a** Evans Blue was intravenously administered and measured in lung homogenates to assess pulmonary vascular permeability; **b** The total protein contents in the BALF were measured using the BCA method to assess protein leakage; **c** The lung weight and body weight ratio were analysed to assess lung oedema; *n* = 3, *P < 0.05; (D) Ultrathin lung sections were scanned using TEM to assess vascular endothelial injury by a blind observer; N indicates nuclear; pentagram indicates bubbling of pyroptotic cells; scale bar = 5 μm

Endothelial injury was the primary contributor to interstitial oedema and alveolar injury in acute lung injury in sepsis. To investigate the effect of HGF on endothelial injury, we measured the pulmonary vascular permeability of the septic mice after the administration of recombinant HGF. As shown in the results of the Evans blue assay, the vascular permeability was higher in the septic mice than in the Sham mice, which was consistent with the protein levels in the BALF (Fig. 2a-b). Compared to CLP alone, intravenous administration of recombinant HGF dramatically decreased the effusion of Evans blue and protein, clearly indicating improved permeability (Fig. 2a-b). Severe pulmonary oedema was also detected in the septic mice, as revealed by lung weight/body weight (LW/BW) ratio, and this pulmonary oedema was abrogated by HGF (Fig. 2c) in the CLP + HGF group. There was no significant difference between the Sham and Sham+HGF groups.

Ultrathin lung sections were scanned by TEM to assess pulmonary vascular endothelial injury. A number of pyroptotic morphological features, such as cytoplasmic swelling, bubbling, osmotic lysis, nuclear condensation and oligonucleosomal DNA fragmentation, appeared in the pulmonary vascular endothelial cells in the ultrathin sections from the septic mice. However, endothelial pyroptosis was mitigated in the septic mice by the administration of HGF (Fig. 2d).

### HGF improved endothelial pyroptosis in vitro

**Fig. 3:**
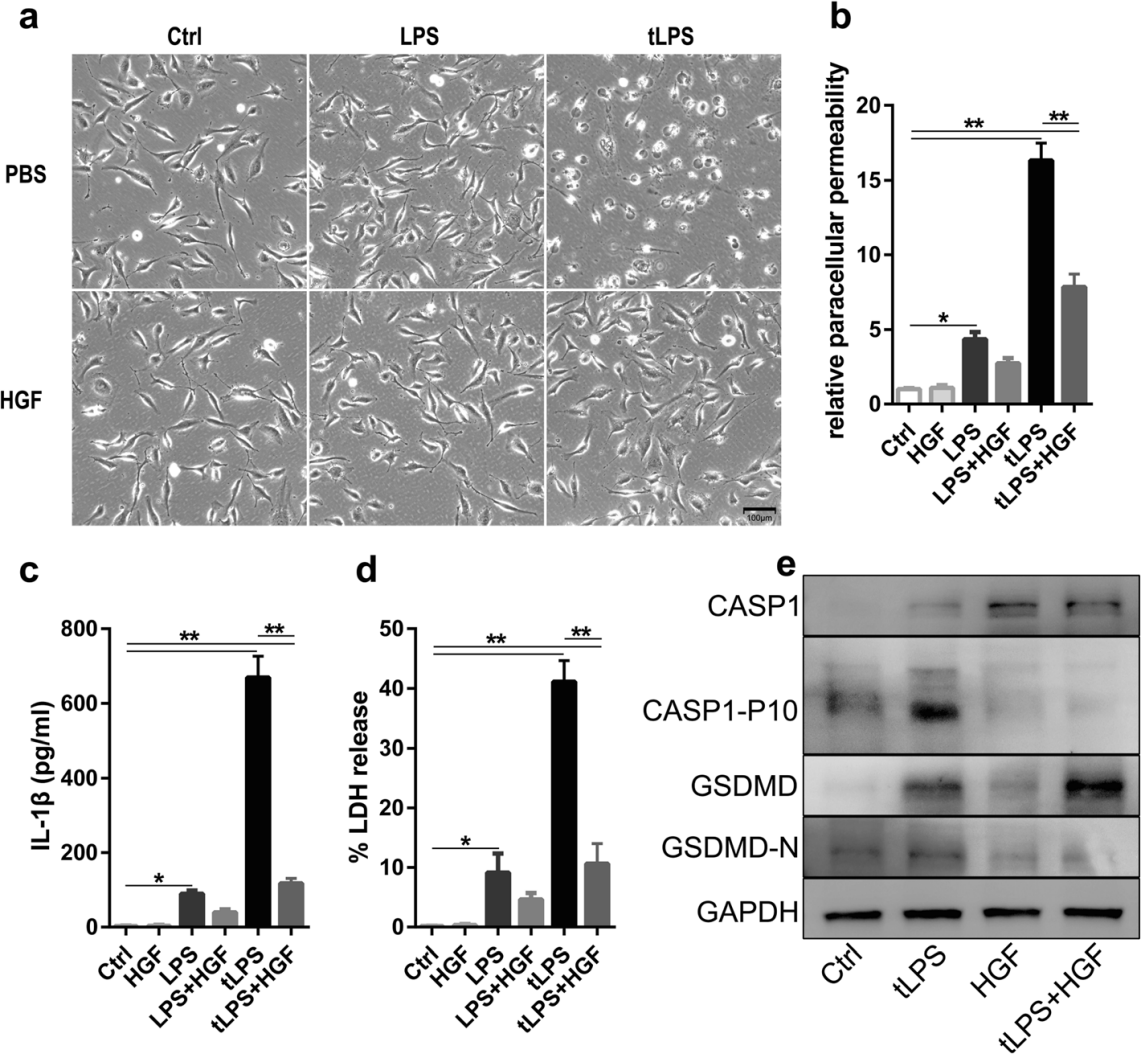
HGF alleviated endothelial pyroptosis in vitro. EA.hy926 cells were stimulated with LPS (2.5 μg/mL) with or without Lipo2000 (2 μL/mL), followed by administration of HGF (25 ng/mL) immediately and 6 h later. **a** Bright-field image of treated EA.hy926 cells from randomly selected fields of view, in which morphological changes characteristic of pyroptosis were assessed; scale bar = 100 μm; **b** Relative paracellular permeability of single-layer EA.hy926 cells exposed to different treatments and analysed by FITC-dextran; **c** IL-1β in the supernatant of treated EA.hy926 cells was measured by ELISA; **d** LDH release assay was performed to measure pyroptosis of the treated EA.hy926 cells, *n* = 3, *P < 0.05, ** *P* < 0.01; **e** CASP-1, CASP-1-P10, GSDMD and GSDMD-N in the treated EA.hy926 cell homogenates were measured by Western blot; *n* = 3

To confirm the effect of HGF on endothelial pyroptosis, umbilical vein endothelial pyroptosis was induced by transfected LPS (tLPS) in vitro. The EA.hy926 cells presented a typical pyroptotic morphology, including cell swelling, membrane rupture, bubbling, and bubble-like cell protrusions, and a large amount of LDH and IL-1β was released into the extracellular milieu after LPS transfection (Fig. 3a-d). LPS stimulation also promoted, to some extent, LDH release and IL-1β secretion by EA.hy926 cells (Fig. 3c-d) but did not induce pyroptosis (Fig. 3a). However, recombinant HGF significantly alleviated pyroptosis and decreased LDH and IL-1β release (Fig. 3a-d). Gasdermin D (GSDMD) and caspase-1 cleavage in endothelial cells were decreased by HGF administration (Fig. 3e). HGF dramatically protected mitochondrial integrity and decreased ROS production in pyroptotic endothelial cells (see Additional file 1 Fig. S1).

### HGF ameliorated endothelial pyroptosis by promoting mTOR signalling

**Fig. 4:**
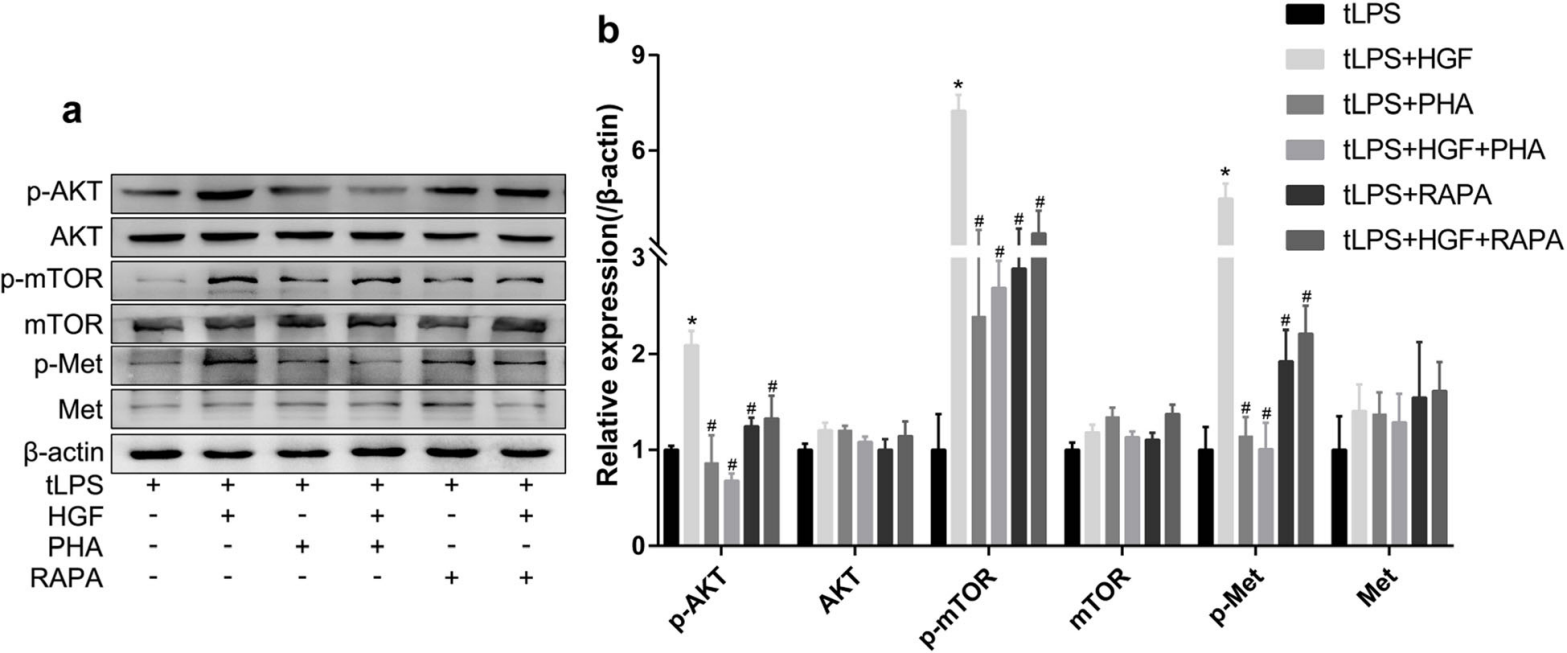
c-Met/mTOR signalling pathway was measured by Western blot. EA.hy926 cells were stimulated with Lipo2000 (2 μL/mL) and LPS (2.5 μg/mL), followed by administration of HGF (25 ng/mL) with or without PHA-665752 (50 nM) or rapamycin (20 nM) immediately and 6 h later. **a** Met, p-Met-Tyr1234/1235, mTOR, p-mTOR-Ser2448, AKT and p-AKT-Ser473 in the treated EA.hy926 cell homogenates were measured by Western blot; **b** Quantification of relative expression, *n* = 3; *P < 0.05 compared with the tLPS group; #*P* < 0.05 compared with the tLPS+HGF group; PHA: PHA-665752, RAPA: rapamycin.

A crucial mechanism of growth factor-induced downstream biological effects is the constitutive activation of mTOR [20]. HGF specifically binds to the receptor c-Met and then activates the key downstream AKT/mTOR signalling pathway to play a crucial role in cell survival and other programmed cell death, such as apoptosis and autophagy [21]. To test whether the c-Met/mTOR signalling pathway mediates the protective role of HGF against endothelial pyroptosis, PHA-665752 (PHA) and rapamycin (RAPA), specific inhibitors of Met and mTOR, respectively, were administered to the endothelial cells. Immunoblotting revealed that c-Met in the plasma membrane was phosphorylated in response to the binding of HGF, and this c-Met phosphorylation was abolished by PHA-665752 in the LPS-transfected endothelial cells (Fig. 4). In addition, HGF treatment dramatically phosphorylated mTOR-Ser2448 and AKT-Ser473, which indicate the activation of mTOR [22], in pyroptotic endothelial cells. However, mTOR activation was abrogated by rapamycin, a specific inhibitor of mTOR, as evidenced by the weak expression of P-AKT-Ser473 and P-mTOR-Ser2448 (Fig. 4).

**Fig. 5:**
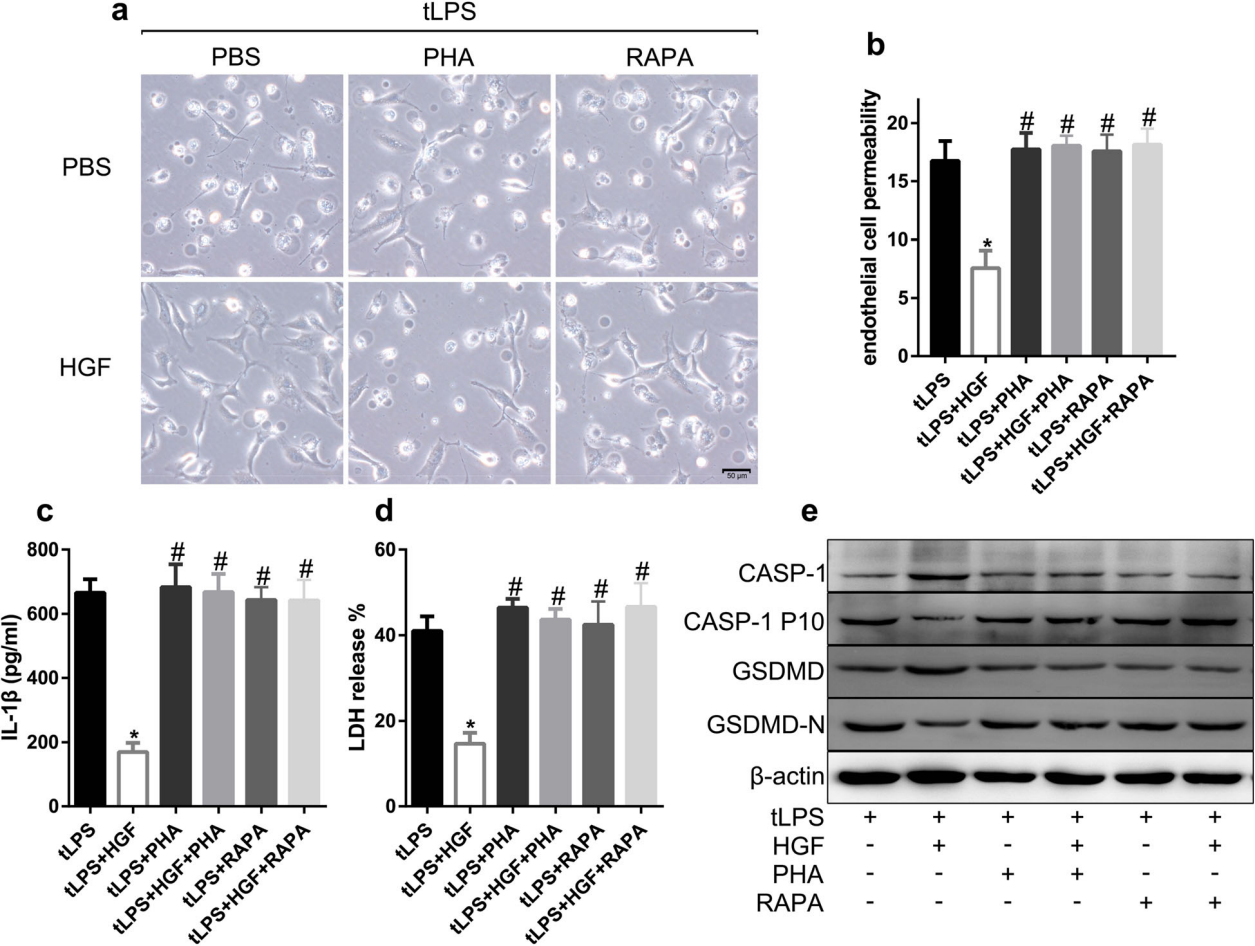
HGF ameliorated endothelial cell pyroptosis by promoting mTOR signalling. **a** EA.hy926 cells were treated as described, and images were captured from randomly selected fields of view by bright field microscopy to assess the morphological changes characteristic of pyroptosis; scale bar = 50 μm; **b** Relative paracellular permeability of single-layer EA.hy926 cells exposed to different treatments was measured by FITC-dextran; **c** IL-1β in the supernatant of treated EA.hy926 cells was measured by ELISA; **d** LDH release assay was performed to measure pyroptosis of treated EA.hy926 cells; **e** CASP-1, CASP-1-P10, GSDMD and GSDMD-N in the treated EA.hy926 cell homogenates were measured by Western blot; *n* = 3, *P < 0.05 compared with the tLPS group; #*P* < 0.05 compared with the tLPS+HGF group; PHA: PHA-665752, RAPA: rapamycin

HGF administration remarkably improved the endothelial cell pyroptosis caused by LPS transfection, as revealed by the morphological features observed by bright field microscopy, and this effect was obviously abolished by the inhibitor of c-Met, PHA-665752 (PHA, 50 nM), and the inhibitor of mTOR, and rapamycin (RAPA, 20 nM) (Fig. 5a). The inhibitory effect of HGF on IL-1β secretion and LDH release by pyroptotic endothelial cells was impaired by PHA-665752 and rapamycin (Fig. 5c, d). Moreover, the inhibition of GSDMD or caspase-1 cleavage by HGF was abolished by PHA-665752 and rapamycin (Fig. 5e). HGF alleviated endothelial cell pyroptosis, and this effect was dependent on the promotion of mTOR signalling.

### HGF protected mitochondrial physiology by promoting mTOR signalling

**Fig. 6:**
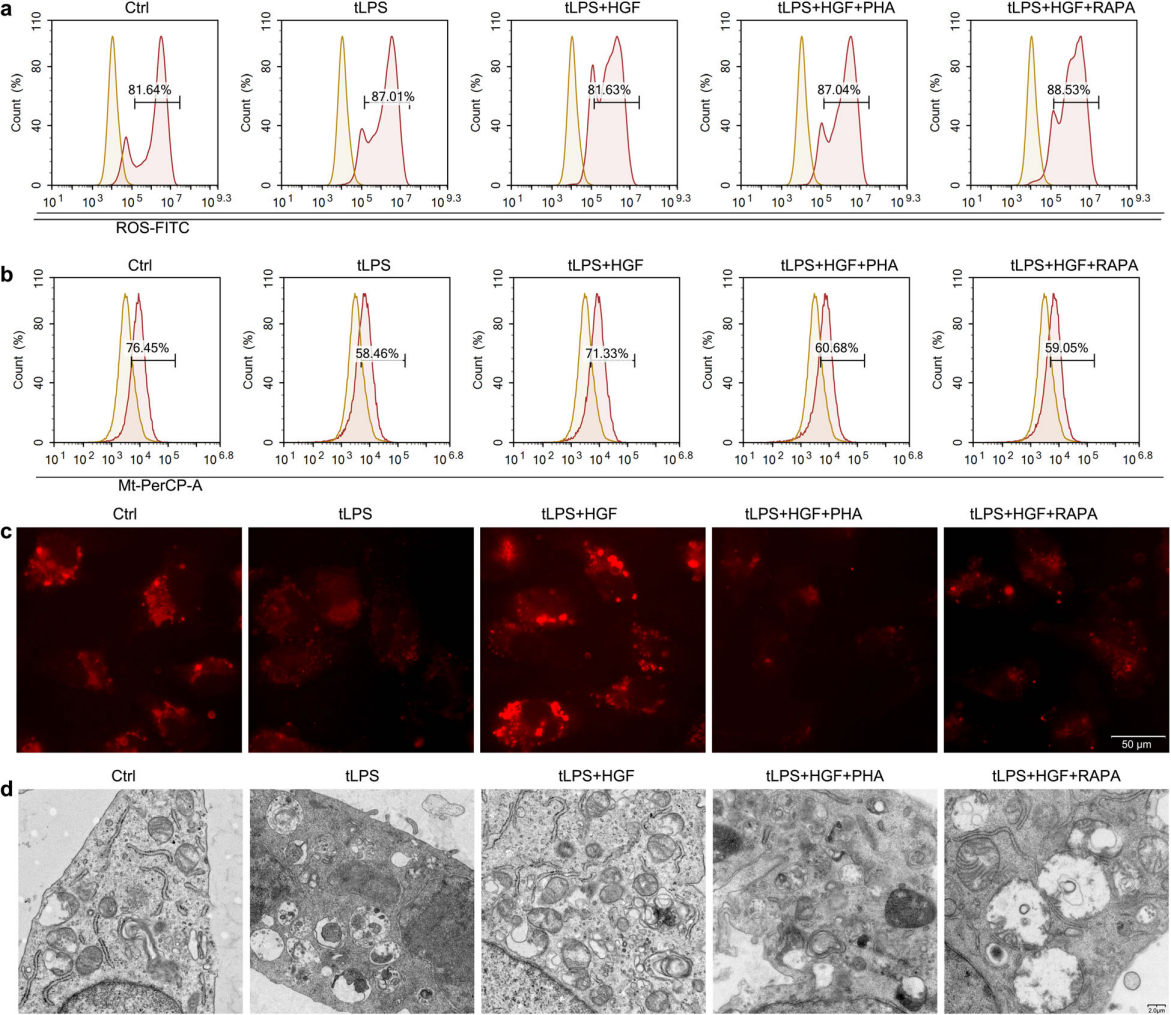
HGF protected mitochondrial physiology by promoting mTOR signalling. **a, b** EA.hy926 cells were treated as described, stained with DCFH-DA and MitoTracker for 30 min, and analysed by flow cytometry; *n* = 3; **c** EA.hy926 cells cultured on glass coverslips were treated as described and stained with MitoTracker for 30 min and photographed using a fluorescence inversion microscope system; scale bar = 50 μm; **d** Ultrathin EA.hy926 cell sections were scanned by TEM to assess mitochondrial injury; scale bar = 2 μm; PHA: PHA-665752, RAPA: rapamycin

Although mTOR signalling was confirmed to govern mitochondrial integrity and function in previous studies [23], whether HGF/c-Met considerably directs crosstalk remains poorly defined. We assessed mitochondrial integrity and function after HGF administration in LPS-transfected EA.hy.926 cells. As the flow cytometry and immunofluorescence results demonstrated, HGF dramatically protected mitochondrial integrity and decreased ROS production in the pyroptotic endothelial cells (Fig. 6a-c). Mitochondrial injury in the tLPS-treated EA.hy926 cells was mitigated by HGF, which was also evidenced by the TEM results (Fig. 6d). However, all the protective effects of HGF were abrogated by the inhibition of mTOR.

## Discussion

The results of our study demonstrated that HGF effectively ameliorates sepsis-induced acute lung injury and pulmonary vascular endothelial injury. The protective role of HGF involves inhibiting endothelial pyroptosis by protecting mitochondrial physiology through the mTOR signalling pathway. To the best of our knowledge, this is the first report that describes the mechanism by which the mTOR signalling pathway plays a key role in the amelioration of endothelial pyroptosis by HGF.

HGF is a multifunctional growth factor that is involved in a variety of physiological activities and pathological processes. Enhancement of endogenous HGF response for minimizing septic tissue injuries, however, secretion of HGF seems insufficient in sepsis, with a time lag between the injury and HGF increasing. Here, we demonstrated that HGF alleviates polymicrobial sepsis-induced lung injury and inflammation and improves the survival rate of septic mice. Previous studies have illustrated that HGF alleviates acute kidney injury and acute hepatic injury in mice with LPS-induced sepsis [24, 25]. Several lines of clinical evidence have demonstrated that blood HGF levels raised in response to organ damage in patients with sepsis in the early phase [8]. Therefore, this experimental result indicates that HGF supplement therapy might be available for improving sepsis-induced organ damage. Endothelial injury and lung injury were not fully reversed by HGF, probably due to the complex pathophysiological mechanism of polymicrobial sepsis and the subsequent injury induced by persistent inflammation, nor did recombinant HGF rescue all of septic mice in previous researches [26]. An efficient dose of HGF (1 μg/g) was chosen to intravenously treat septic mice immediately and at 12 h after CLP operation, because the lung is the most susceptible organ. Kamimoto M et al. [27] reported HGF treatment was gaven twice 1 day after LPS challenge 0 and 12 h. Kosai K et al. [26] gave recombinant HGF intraperitoneally injections 6 h before and 3 h after LPS injection, because only the few hours from liver injury to death. However, for clinical treatment of sepsis with HGF, clinical studies are necessary, the dose and timing of HGF administration should be determined according to its pharmacokinetics and pharmacodynamics in patients. Therefore, HGF may be a promising supplemental therapy to ameliorate sepsis-induced organ damage.

Endothelial pyroptosis is a vital mechanism of vascular endothelial injury in sepsis, and this process leads to the release of a plethora of pro-inflammatory cytokines, such as IL-1β and LDH, and destroys the endothelium barrier, eventually leading to septic shock and multiple organ failure [13, 14, 28]. The specific inhibition of endothelial pyroptosis attenuates LPS-induced ALI and decreases sepsis-induced mortality in *Casp11*^*EC−/−*^ mice [9], which means that endothelial pyroptosis is a promising therapeutic target. The present study illustrated that HGF effectively inhibited endothelial pyroptosis, reduced vascular permeability, and decreased IL-1β and LDH secretion. Although previous studies have shown that HGF has anti-apoptotic and anti-necrotic effects, this is the first study reporting its anti-pyroptotic effect, which is meaningful for dissecting the mechanism by which HGF repairs endothelial injury.

Mitochondrial damage is a crucial contributor to and hallmark of pyroptosis [29, 30]. Many stress factors, such as microbiome metabolites, toxicants and oxidized microenvironments, have been shown to disrupt mitochondrial homeostasis [31]. In addition, the gasdermin pore in the plasma membrane eventually executes pyroptosis, simultaneously causing the mitochondria to release its contents [32]. The ROS, mtDNA, and ATP released from injured mitochondria strongly promote pyroptosis by activating the inflammasome and the cleavage of caspase-1 [33–35]. Our results have shown that HGF protects the integrity of the mitochondrial plasma membrane, reduces the release of mitochondrial contents, leads to the scavenging of ROS or other mitochondrial damage-associated molecules and prevents pyroptosis [36, 37]. Thus, improving mitochondrial physiology alleviates endothelial pyroptosis and may be a therapeutic target for sepsis.

HGF binds to c-Met in the plasma membrane, activates the AKT/mTOR signalling pathway, plays a vital role in cell growth, metabolism, cell survival and migration; in addition, HGF is closely associated with developmental defects, cancer, diabetes and autoimmune diseases [22, 38]. Our results demonstrated that HGF activates the AKT/mTOR signalling pathway to protect mitochondrial physiology and reduce pyroptosis in endothelial cells. Previous studies have proven that mTOR controls the structure and function of mitochondria. mTOR complex 1 selectively promotes the translation of nucleus-encoded, mitochondria-related mRNAs to control mitochondrial activity and biogenesis [39]. mTOR complex 2 localizes to the plasma membrane of mitochondria to mediate its integrity and control mitochondrial physiology [23]. Thus, mTOR signalling appears to be a particularly important hub for HGF in the repair of endothelial injury. Other downstream pathways of HGF/c-Met, such as the MAPK, Ras/MEK, STAT3, IκBα/NF-κB pathways, were reported to mediate invasive growth, resist apoptotic insults and cause proliferattion. Although our previous study [15] revealed that following HGF stimulation, STAT3 was activated and endothelial apoptosis partially attenuated, we did not measure the effects of these pathways on endothelial pyroptosis here, which is a limitation.

In summary, we demonstrated that mTOR signalling mediates the protective effect of HGF on mitochondrial physiology. HGF inhibits the release of mitochondrial contents to alleviate endothelial pyroptosis in vitro. The effect of HGF in attenuating vascular endothelial injury could alleviate acute lung injury in sepsis and improve the prognosis of sepsis, at least in animal model. Although the effect of HGF on clinical sepsis patients is speculative and remains to be elucidated, it is tempting to believe that it could be a promising adjuvant therapy for sepsis.

## Conclusion

HGF ameliorates endothelial pyroptosis depending activation of mTOR signalling by protecting mitochondrial physiology, avoiding mitochondrial damage-associated molecular release in vitro. The recombinant HGF intravenously administration mitigates polymicrobial sepsis-induced pulmonary vascular endothelial pyroptosis, attenuates pulmonary vascular endothelial injury and acute lung injury in mice. HGF may have the potential to be a promising adjuvant therapeutic strategy aimed at the treatment of sepsis and acute lung injury.

## Supplementary information

**Additional file 1: Figure S1.** HGF alleviated endothelial pyroptosis *in vitro*. EAhy926EA.hy926 cells were stimulated with LPS (2.5ug/mL), lipo2000 (2uL/mL) and LPS (2.5ug/mL) for 6h, followed by HGF administration (25ng/mL) 6h, respectively. EA.hy926 cells were treated as mentioned and stained with DCFH-DA and MitoTracker for 30min, measured by flow cytometry; *n* = 3.

## Data Availability

Figure 1S HGF protected mitochondrial physiology in vitro. EA.hy926 cells were treated as described, stained with DCFH-DA and MitoTracker for 30 min, analysed by flow cytometry; *n* = 3.

## Abbreviations

Term: Definition
HGF: Hepatocyte growth factor
TEM: Transmission electron microscopy
LPS: Lipopolysaccharide
ROS: Reactive oxygen species
DAMP: Damage-associated molecular patterns
MSC: Mesenchymal stem cell
CLP: Caecal ligation puncture
LDH: Lactate dehydrogenase
BALF: Bronchoalveolar lavage fluid
ALI: Acute lung injury
LW/BW: Lung weight/body weight
tLPS: Transfected LPS
GSDMD: Gasdermin D
BCA: Bicinchoninic acid method
RAPA: Rapamycin
mTOR: Mammalian target of rapamycin
