# Supplementary material for: HGF alleviates septic endothelial injury by inhibiting pyroptosis via the mTOR signalling pathway
Source: Respir Res. 2020 Aug 14;21:215. doi: 10.1186/s12931-020-01480-3 (PMC7427898; doi:10.1186/s12931-020-01480-3)
Supplement: Supplementary file 1 — Additional file 1: Figure S1. HGF alleviated endothelial pyroptosis in vitro. EAhy926EA.hy926 cells were stimulated with LPS (2.5ug/mL), lipo2000 (2uL/mL) and LPS (2.5ug/mL) for 6h, followed by HGF administration (25ng/mL) 6h, respectively. EA.hy926 cells were treated as mentioned and stained with DCFH-DA and MitoTracker for 30min, measured by flow cytometry; n = 3. [file 12931_2020_1480_MOESM1_ESM.docx]

Additional method:

Acute lung injury scoring system:

Six visual fields of histopathological section were randomly photographed and scored as following by five criteria. Edema, alveolar and interstitial inflammation, alveolar and interstitial hemorrhage, atelectasis, and hyaline membrane formation were each scored on a 0‐ to 4‐point scale: no injury = score of 0; injury in 25% of the field = score of 1; injury in 50% of the field = score of 2; injury in 75% of the field = score of 3; and injury throughout the field = score [Smith KM, et al. Crit Care Med. 1997; 25(11): 1888-97.].

Additional result:


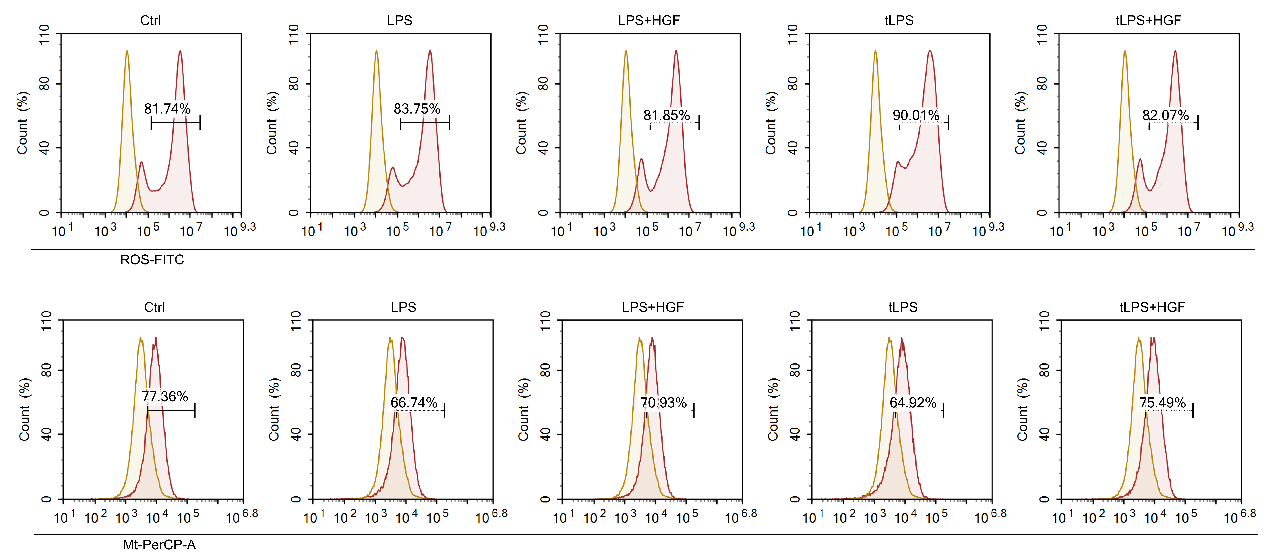


Figure S1. HGF alleviated endothelial pyroptosis *in vitro*. EA.hy926 cells were stimulated with LPS (2.5μg/mL), lipo2000 (2μL/mL) and LPS (2.5μg/mL) for 6h, followed by HGF administration (25ng/mL) 6h, respectively. EA.hy926 cells were treated as mentioned and stained with DCFH-DA and MitoTracker for 30min, measured by flow cytometry; n = 3.
